# Livestock-associated MRSA survival on house flies (*Musca domestica*) and stable flies (*Stomoxys calcitrans)* after removal from a Danish pig farm

**DOI:** 10.1038/s41598-021-83228-7

**Published:** 2021-02-11

**Authors:** Jonno Jorn Stelder, Lene Jung Kjær, Lars Bogø Jensen, Anette Ella Boklund, Matt Denwood, Margrethe Carlsen, René Bødker

**Affiliations:** 1 Section for Animal Welfare and Disease Control, Department of Veterinary and Animal Sciences, Copenhagen University, Grønnegårdsvej 8, 1870 Frederiksberg C, Denmark; 2 National Food Institute, DTU Technical University of Denmark, Kemitorvet Building 204, 2800 Lyngby, Denmark

**Keywords:** Ecological epidemiology, Infectious diseases

## Abstract

We caught stable- and house flies on a Danish LA-MRSA positive pig farm. Stable- and house flies were housed together and culled over time to test for the presence of live LA-MRSA bacteria at 24 h intervals to establish the length of time for which LA-MRSA can persist on flies. On average, 7% of stable flies and 27% of house flies tested positive for LA-MRSA immediately upon removal from the farm. LA-MRSA prevalence decreased over time and estimates based on a Kaplan–Meier time-to-event analysis indicated that the probability of a stable- or house fly testing positive for LA-MRSA was 5.4% and 7.8% after 24 h, 3.5% and 4.3% after 48 h, 3.1% and 2.2% after 72 h and 0.4% and 0% after 96 h of removal from the pig farm, respectively. Simultaneously, we found that caged cultivated house flies became carriers of LA-MRSA, without direct contact with pigs, in the same proportions as wild flies inside the farm. We provide distance distributions of Danish pig farms and residential addresses as well as the calculated maximum dispersal potentials of stable- and house flies, which suggest that there is a potential for stable- and house flies dispersing live LA-MRSA bacteria into the surrounding environment of a pig farm. This potential should therefore be considered when modelling the spread between farms or the risk posed to humans living in close proximity to LA-MRSA pig farm sources.

## Introduction

The emerging Methicillin-resistant strain of *S. aureus* associated with livestock (LA-MRSA), has seen a rapid increase and spread amongst pig farms in recent years^1^, particularly in the Netherlands^2^ as well as in Denmark^3^. In 2014, 68% of Danish pig farms tested positive for LA-MRSA, further increasing up to 88% by 2016^4^. Although several pathways have been described that contribute to the spread of LA-MRSA, simulation models based on the known pathways for the spread of LA-MRSA between pig farms are not able to explain the entire observed spread in Denmark^5^. In humans, the number of infections seems to have reached a stable level in 2014^6,7^. Interestingly, the vast majority of LA-MRSA cases in humans concern people from rural areas as opposed to urban areas. In many of these rural cases, there is an absence of direct contact between infected people and livestock^1,8^ so spread might have occurred by different routes. MRSA bacteria can spread downwind from pig farms into the surrounding environment^9^ in levels that could potentially pose a health risk to humans^10^. However, due to the spread pattern of human infection cases in rural areas with a high density of pig farms, Anker et al.^1^ suggest that the cause of these infections cannot entirely be attributed to environmental spread and that other transmission pathways must be contributing to this.

Due to their hematophagous and saprophytic foraging behaviours, as well as their breeding behaviour, stable flies and house flies, respectively, play a significant role in the spread and transmission of a wide variety of bacterial and viral pathogens. Pathogens present in the blood or on the skin of livestock or humans can be mechanically transmitted by stable flies that collect live bacteria on their exoskeleton during foraging^11^, while house flies obtain pathogens either through foraging on or direct contact with septic substrates, which they can subsequently mechanically transmit ^12–14^. Some of the known pathogens that stable- and house flies can transmit are *Chlamydia trachomatis*^15^, *Campylobacter jejuni*^16^, African swine fever virus and capripox virus^17^ as well as *Staphylococcus aureus*^18^. House flies were found to be capable of picking up infectious chlamydial particles on their proboscis or legs, with which they could transmit trachoma infections for several hours from host to host^15^. The same was found for *Campylobacter* bacteria, where house flies that were inoculated via their proboscis tested positive for over 24 h as *C. jejuni* levels decreased with time^16^. Regular St*aphylococcus aureus* can be transmitted by house flies up to 6 h, with a peak between 0–2 h, post-ingestion through the excretion of viable bacteria, making them important vectors^18^. Stable flies can become mechanical vectors of capripox virus up to 24 h post-ingestion, as well as African swine fever for up to 48 h and possibly longer when taking a bloodmeal from an infected host^17^. We found no studies that have specifically investigated the role of flies in the dispersal of LA-MRSA, despite their known vector competency for a range of bacterial pathogens. However, based on the examples of different pathogens, we suggest that by carrying live bacteria on the legs, proboscis or exoskeleton by both stable- and house flies, transmission of LA-MRSA is also a possibility. Stable- and house flies exhibit hematophagous and saprophytic foraging behaviours, respectively, actively seeking the places on a host which accommodate high levels of LA-MRSA bacteria, such as the skin^19^ (in the case of stable flies) and mucous membranes like the nasal area^19–21^ (in the case of house flies). Given these foraging behaviours, this potential transmission pathway could be relevant as flies, contrary to windborne environmental spread, are able to actively target a host, a mucosal surface or a skin lesion.

Stable flies are known to recolonize farms locally in spring and early summer^22^, which is evidence of migration occurring between pig farms. In this study, we quantify the plausibility of a potential LA-MRSA transmission pathway through stable- and house flies as well as its dispersal potential. In this study, the dispersal potential is expressed as the function of dispersal distance per time (flight speed) of a fly and the survival time of LA-MRSA bacteria externally present on the fly (survival rate). However, it should be noted that the actual dispersal distance is most likely shorter than our estimated dispersal potential, as flight is likely to be interspersed by many behavioural and environmental factors. Therefore, the dispersal potential should be regarded as the maximum distance a fly could travel while carrying live LA-MRSA bacteria. The purpose of this study was to assess the prevalence of LA-MRSA amongst the stable- and house fly populations of a Danish pig farm and to quantify how long these LA-MRSA bacteria on the exoskeleton of flies survive after being removed from a pig farm source. By doing so, we found a practical approach to estimate the temporal factor in the dispersal potential of these two fly species.

Stable- and house flies are capable of covering large distances^14,23,24^, although estimates on exact dispersal distances and proportions vary widely. We can use the different dispersal and flight distances from previous studies to make rough estimates of how far stable- and house flies could potentially fly while carrying live LA-MRSA bacteria. Using these previous studies, we also reflect on the distance distribution between pig farms and residential addresses in Denmark, as well as the corresponding potential of stable- and house flies to cover these distances while carrying live LA-MRSA bacteria. This is because transmission of LA-MRSA between pig farms or from pig farms to residential addresses through stable- and house flies is only possible if the flies are capable of traversing these distances while these bacteria are still alive. The relatively small size of Denmark and the high density of pig farms could also make this potential transmission pathway more viable in Denmark compared to other countries.

In this study, we establish only the binary presence/absence of bacteria rather than quantifying the number of bacteria present on the flies over time. Therefore, we limit our discussions to dispersal potential rather than mechanical vector potential. We ran a subsidiary experiment to establish whether direct contact with a pig or a pig farm’s interior surfaces is required for a fly to become a carrier of LA-MRSA bacteria, or if airborne LA-MRSA particles alone suffice in the contamination of flies.

## Materials and methods

### Study site

We carried out the study in June and July 2019, on a single high-biosecurity (Danish Specific Pathogen Free system^25^) Danish pig farm with around 450 sows, situated on Zealand (Sjælland). We selected a room housing 10–20 sows on average for the first experiment due to its relatively stable conditions in terms of fly population composition and LA-MRSA prevalence. For the second experiment, we also included an adjacent piglet room housing around 40 piglets.

### Fly collection

We caught approximately 300 stable- or house flies per collection date between 10:00–12:00 each time from inside the farm. This number provided a sufficient sample size, while also being logistically feasible to handle and process. We excluded other fly species, either due to their low numbers or their apparent incapacity to move between farms. We used butterfly nets and plastic cups to catch both fly species at the same time. We placed the stable- and house flies together into 12 cylindrical plastic containers with mesh netted ends, carrying approximately 25 flies each. There was little variation in the relative proportion of each species inside the containers throughout the experiment, with the majority of flies inside the containers being stable flies, averaging at 75% ± 5.5% (95% CI) relative to house flies. Most flies were caught using the butterfly nets by sweeping it along the flanks of pigs, while the remainder of flies needed to get the desired number of flies per container, were caught while resting on the walls of the stable using plastic cups. The butterfly nets and plastic cups were occasionally renewed when deemed necessary due to accumulated filth or holes, and they were stored away from the pigs or flies in-between collection dates in an attempt to let any remaining LA-MRSA bacteria perish over time.

### Laboratory conditions

We used a non-climate controlled microbiology laboratory at the National Food Institute, Technical University of Denmark (DTU), to keep the cylindrical fly containers at room temperature and to test the flies for LA-MRSA presence. The flies were fed every 24 h by placing sugar water-soaked (1:3 sugar to water ratio) cotton pads on the outside of the cylindrical containers, on top of the mesh.

### LA-MRSA survival time

We culled subsamples of the total 300 flies caught at set time intervals and tested them for the presence of LA-MRSA. At each time interval of 24 h, we culled the flies from 2 cylindrical containers selected at random, using CO_2_ gas from a SodaStream machine modified with a rubber nozzle to fill zip lock bags containing the cylindrical containers. Before culling, we removed dead flies using an aspirator, to make sure that all the flies used in the analysis were alive at the time of culling. We did this at the times of 0, 24, 48, 72 and 96 h after collection of the flies at the farm and arrival at the laboratory. On one occasion (24-06-2019), due to logistical reasons, we had to cull a sample 21 h after collection rather than 24 h. In total, we collected flies on 5 dates: 24-06, 08-07, 15-07, 22-07 and 29-07. For the first time interval at 0 h after capture, we culled the flies inside 4 of the cylindrical containers and tested for LA-MRSA to establish a baseline prevalence of LA-MRSA. For each of the remaining time intervals, we subsampled the flies inside 2 cylindrical containers. We increased the subsample size at the first time interval to establish a baseline prevalence of LA-MRSA for that collection date. In order to identify the presence of LA-MRSA bacteria, we used a similar technique as Hansen et al.^26^, although slightly modified to better suit our study. We placed the culled flies individually into microtiter plate wells (BioLite 48 wells Multidish) containing 0.5 ml Mueller–Hinton 6.5% NaCl liquid media and stirred gently. Subsequently, we placed the microtiter plates in an incubator for 18 h at 37 °C. Once incubated, we took samples of each well using 1 µl inoculation loops which we streaked out onto MRSA-selective agar plates (Oxoid Brilliance MRSA 2 AGAR). We placed the agar plates into an incubator again for another 18 h at 37 °C. Afterwards, we screened the agar plates visually for LA-MRSA, and used Matrix-assisted laser desorption/ionization time-of-flight (MALDI-TOF) mass spectrometry if clarification was needed^27^.

### LA-MRSA environmental contamination

To investigate whether flies could become contaminated with LA-MRSA through exposure to environmental aerosols or airborne dust inside the pig farm, we obtained 800 cultivated house fly pupae from the Department of Agroecology at Aarhus University, which we divided over 20 plastic cages (16 × 13 × 12cm) covered with 1mm^2^ plastic mesh walls. We fitted each cage with a small petri dish containing sawdust to aid hatching, as well as a small petri dish containing a wad of cotton soaked in sugar water (1:3 ratio sugar to water). After one week at room-temperature, the majority of flies were hatched and we removed the remaining pupae using an aspirator. We placed the cages inside the pig farm for 48 h, suspended in mid-air above the pig pens at 1.5–2.5 m above the ground. Of the 20 cages, we placed 8 cages in a room containing only sows and placed another 8 cages in a room containing only piglets. We used the remaining 4 cages as controls and kept them at the University of Copenhagen until testing. After 48 h, we caught a combination of free-roaming stable- and house flies approximating a total of 100, which had the same relative proportions as in the previous experiment, native to the indoor stable environment with direct contact to pigs or other surfaces, inside each of the 2 rooms using sweeping nets. We did this to establish a baseline of LA-MRSA prevalence amongst the native fly population in both rooms at the time. No pigs were handled throughout the experiment. We divided the free-roaming flies into 8 clean plastic cylindrical containers (4 per room). In the laboratory at DTU, we culled all the flies using CO_2_ gas and tested them for LA-MRSA using the method described previously.

### Ethics approval and consent to participate

Not applicable since only insects were used in the study.

## Data analyses

### LA-MRSA survival time

We fitted a binary logistic regression model to the data at 0 h after capture using IBM SPSS Statistics (v.25)^28^ to investigate whether the species of fly or collection date had a significant effect on the probability of a fly testing positive for LA-MRSA.

We calculated the overall proportion of flies that tested positive for LA-MRSA at each sampling time point, along with the binomial proportion confidence intervals. We calculated the binomial proportion confidence intervals (Clopper–Pearson^29^) by running a one-sample binomial test in IBM SPSS Statistics.

We analysed the presence/absence of LA-MRSA corresponding to each time point at fly level by creating an interval-censored Kaplan–Meier time-to-event curve separated for each collection date within each fly species. We implemented the models using the “survfit” function within the “survival” package^30,31^ for R (v.3.6.1.)^32^. Following, we used the restricted mean survival and confidence intervals based on these analyses to establish the time after removal from a pig farm source at which LA-MRSA can be found on the exoskeleton of a fly. We used the interval censoring to right censor the flies that tested positive for LA-MRSA at their respective time of culling (i.e. the ‘event’ of the fly no longer being LA-MRSA positive has not occurred yet), and left censor the flies that tested negative for LA-MRSA (i.e. the ‘event’ of the fly no longer being LA-MRSA positive occurred before the fly was tested).

### LA-MRSA environmental contamination

We used Chi-square to test for differences in observed LA-MRSA prevalence between wild free-roaming flies and caged cultivated house flies for each room, as well as between room for each species. To assess any differences in the predicted prevalence of LA-MRSA between the sow room and piglet room for the caged cultivated flies, we used a generalized linear mixed-effects logistic model using the “lme4” package^33^ in R (v.3.6.1)^32^ with cage as a random factor. Since cage was not a factor for the free-roaming flies, we fitted a regular generalized linear model to test for differences in LA-MRSA prevalence between rooms.

### Distance distribution

To assess the distance distribution of both pig farms and residential addresses, we calculated the distance of each Danish pig farm (coordinates in UTM zone 32 N, obtained from the Danish Central Husbandry Register) to its nearest neighbouring pig farm, as well as the distance from each Danish residential address (obtained from the Danish KMS Address Register^34^) to its nearest pig farm. For the residential addresses, we excluded the house closest to a pig farm as this house is usually adjacent to the farm or on the same address.

### Dispersal distances

To make rough estimations of what proportion of flies can fly a certain distance from their farm of origin, while remaining positive for live LA-MRSA, we applied the estimates from our Kaplan–Meier curves to previous published studies on dispersal distances for stable- and house flies.

The literature on flight or dispersal distances of stable- and house flies varies greatly in the distances they present, as well as the timespans over which the data was gathered. We re-calculated flight distance estimates for each species calculated on the basis of one dispersal study on stable flies and three studies on house flies. If no timespan is given in these published studies, we assumed the average lifespan of stable- or house flies as the denominator for our daily flight distance calculation, as well as similar dispersal distances each day. In a temperate region such as Denmark, adult house flies live up to 14–21 days during normal summers^35^, whereas stable flies within an organic dairy farm in Denmark live between 4–11 days^36^.

A mark-recapture study by Taylor et al.^37^ gave detailed data for stable fly dispersal. Their model reported dispersal distances over 7 days in a field, with a mean dispersal of 1.6 km and a maximum of 7.4 km. This translates into 0.23 km/day and 1.06 km/day, respectively, assuming similar dispersal distances each day.

In another mark-recapture study, Quarterman et al.^38^ recovered house flies up to 8 km from their release point within 24 h in rural Georgia, USA, where they noted that the flies tended to seek out favourable foraging and breeding sites. Schoof et al.^39^ found that house fly dispersal is lower in metropolitan areas, recording distances of up to 4.8 km within 48 h (2.4 km every 24 h, assuming similar dispersal distances each day), with the majority of flies not dispersing more than 1.6 km from their release site.

For house flies, Chakrabarti et al.^40^ found that house flies from commercial farms within a ~ 125 km radius of Kansas, USA, were capable of dispersing into the city. Since no timespan for this dispersal distance is given, this distance would translate to a minimum of 5.9–8.9 km per 24 h on average, when divided by the lifespan of adult house flies.

## Results

### LA-MRSA survival time

The binary logistic regression model showed that the initial prevalence of flies that tested positive for LA-MRSA varied significantly at 0 h after collection from the farm. We also found a significantly higher odds ratio (OR = 5.550, *p* < 0.001) in the model of a house fly testing positive for LA-MRSA compared to a stable fly. The binary logistic regression models correctly classified 90.4% of all cases, and accounted for 15.5% (Nagelkerke R^2^) of the variance in LA-MRSA presence amongst the sampled flies.

**Figure 1:**
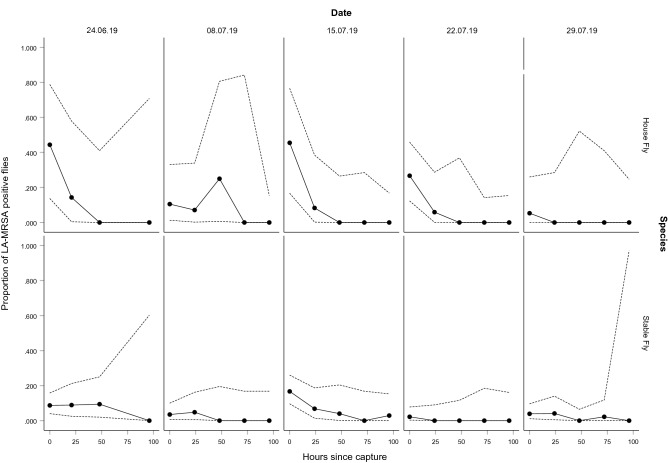
The proportion of house flies and stable flies that tested positive for LA-MRSA from each sampling container and their binomial proportion confidence intervals (95%). The average proportion of LA-MRSA positive house flies (H) and stable flies (S) is 0.2648 (H, n = 18) and 0.07 (S, n = 94) after 0 h, 0.0712 (H, n = 12) and 0.0492 (S, n = 44) after 21/24 h, 0.05 (H, n = 7) and 0.0268 (S, n = 32) after 48 h, 0 (H, n = 11) and 0.0055 (S, n = 26) after 72 h, 0 (H, n = 16) and 0.0058 (S, n = 23) after 96 h.

The proportion of flies that tested positive for LA-MRSA at each time point is illustrated in Fig. 1 for each species. It is important to note the low sample sizes of house flies in the study, which are also reflected by the wide binomial proportion confidence intervals throughout, for each collection date. Also, for both species, sample sizes that were tested for LA-MRSA decreased over time for each collection date, as some flies in each sampling container died during the 96 h.

**Figure 2:**
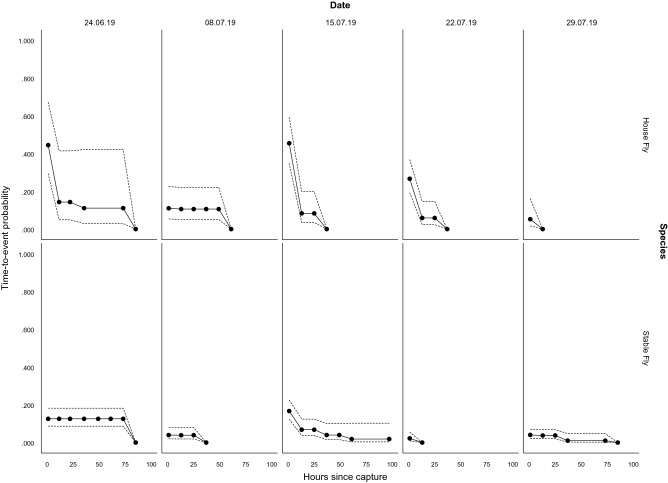
Kaplan–Meier time-to-event curves with confidence intervals (95%) for house- and stable flies for 5 different collection dates and the following 96 h of externally present live LA-MRSA bacteria. As a result of the censoring in the time-to-event model, estimations of the time-to-event probabilities in-between our sampling points are also given.

A Kaplan–Meier curve showing the probability of detecting LA-MRSA at each time point is shown in Fig. 2. We also considered an alternative model using a parametric survival analysis with a Weibull response and found strong effects of collection date and fly species, most likely due to varying starting prevalences of LA-MRSA between collection dates. However, since the data fit the Weibull model poorly, we decided to base the results on the simpler Kaplan–Meier time-to-event curves (fitted separately for each species and collection date) rather than a full survival model (Fig. 2). We also note that the output of these models cannot be interpreted as purely ‘survival’ of LA-MRSA on the flies, as it is in fact a combination of the (varying) initial prevalence of LA-MRSA in each collection date group combined with survival of LA-MRSA over time.

The Kaplan–Meier curves show the decline of LA-MRSA positive flies over time for each species and collection date (Fig. 2). The graphs show that the probability of finding LA-MRSA on house flies drops quickly and then levels out. The probability of finding LA-MRSA on stable flies is more stable over time. Apart from one case, where a single stable fly still tested positive 96 h after capture on the 15th of July, no flies tested positive for LA-MRSA at this time point.

### LA-MRSA environmental contamination

**Figure 3:**
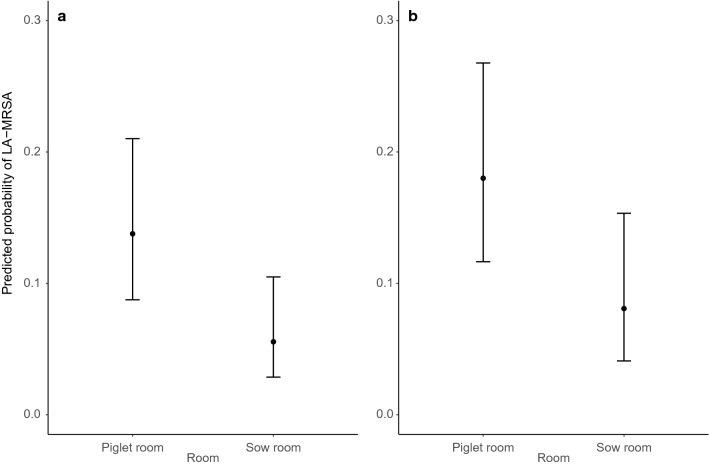
Predicted LA-MRSA prevalence with confidence intervals (95%) between the samples from the piglet room and sow room calculated by the generalized linear mixed-effects logistic model for both caged cultivated house flies (**a**) and calculated by the regular generalized linear model for wild free-roaming flies (**b**). Both the wild free-roaming flies and caged cultivated house flies from the piglet room tested positive for LA-MRSA in significantly greater proportions than the wild free-roaming flies and caged cultivated house flies from the sow room.

The Chi-square test using observed LA-MRSA prevalence numbers indicated that there was no significant difference in observed LA-MRSA prevalence between the caged cultivated house flies and wild free-roaming flies in both the piglet (*p* = 0.432) and sow room (*p* = 0.491) (Fig. 3).

Both the generalized linear mixed-effects logistic model on the caged cultivated house flies and the generalized linear model on wild free-roaming flies showed significant differences in the predicted LA-MRSA prevalence between the sow room and the piglet room (*p* = 0.018 and *p* = 0.43 respectively, Fig. 3). For the cultivated caged house flies, the random factor had a variance of 0.174 in the logistic model, indicating that environmental LA-MRSA bacteria in the air are not uniformly spread within each of the two rooms.

### Distance distribution

**Figure 4:**
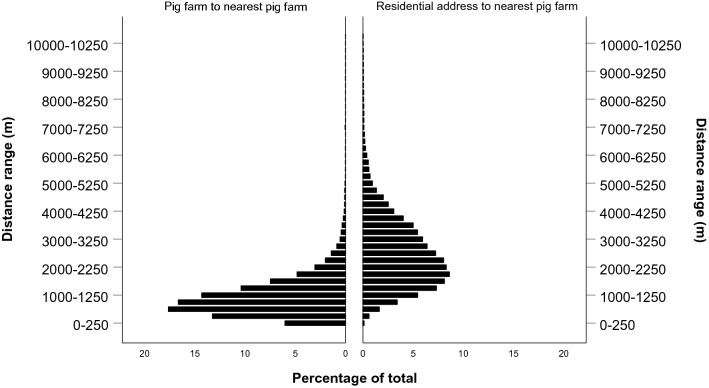
Distance distribution of all Danish pig farms (n = 7569) to their nearest neighbouring pig farm, along with the distance distribution of all Danish residential addresses (n = 2,138,893) to their nearest pig farm. The graph displays 100% of Danish pig farms and 99.1% of residential addresses found in Denmark. Each bar represents a range of 250 m.

The population pyramid showing the distance distribution between Danish pig farms and their nearest neighbouring pig farm as well as residential addresses and their nearest pig farm is shown in Fig. 4. The population pyramid shows that 35.2%, 65.2% and 96.2% of residential addresses in Denmark (n = 2,057,350) are within 2, 3 and 6 kms of a pig farm respectively. For pig farms and their nearest neighbouring pig farm (n = 7562), these numbers are 90.1%, 97.9% and 99.9% for 2, 3 and 6 kms, respectively.

### Dispersal distances

Given the dispersal ranges from Taylor et al.^37^, stable flies would be able to traverse the distance between 0.1–5.8% of residential addresses and their nearest pig farm within 24 h, 0.7–43.5% within 48 h and 2.4–71.1% within 72 h. From a random pig farm, stable flies would be capable of reaching the nearest neighbouring pig farm in 6.0–53.5% of cases within 24 h, 19.3–90.5% of cases within 48 h and 36.9–98.4% of cases within 72 h.

With the daily flight distances found by Quarterman et al.^38^, house flies would be able to traverse the distance between 98.7% of residential addresses and their nearest pig farm within 24 h. From a random pig farm, house flies would be capable of reaching the nearest neighbouring pig farm in 99.9% of cases within 24 h.

With the adjusted daily dispersal distances in metropolitan areas from the Schoof et al.^39^ study, house flies would be able to traverse the distance between 51.6% of residential addresses and their nearest pig farm within 24 h, 93.2% within 48 h and 98.4% within 72 h. From a random pig farm, stable flies would be capable of reaching the nearest neighbouring pig farm in 95.6% of cases within 24 h and 99.7% of cases within 48 h.

The average daily dispersal distance estimated from Chakrabarti et al.^40^ would allow house flies from a random Danish pig farm reach their nearest neighbouring pig farm in 99.9% of cases, while 97.3–98.9% of residential addresses find at least one pig farm within a 24 h range for a house fly to traverse.

## Discussion

We found that, based on our time-to-event analysis (Fig. 2), the probability of detecting LA-MRSA on both stable- and house flies dropped to zero within 96 h after removal from the farm, with the exception of a single stable fly that still tested positive after 96 h. With most collection dates, we saw a substantial decline in the probability of detecting LA-MRSA within 24 h after removal from the farm. After this, for half of the collection dates, the remaining LA-MRSA decayed within the next 24 h, while for the other half of the collection dates, we saw the remaining LA-MRSA lingering. This gradual decrease indicates that the LA-MRSA bacteria do not replicate on the exoskeleton of the flies, or that the flies shed the bacteria through, for instance, self-grooming or movement. Similar declines of bacteria on house flies have been found in *Campylobacter spp.*^16^, as well as the substantial initial decrease followed by a slower gradual decrease^41^. These *Campylobacter spp.* studies, however, only tested flies up to 24 h after being exposed to the bacteria. In our study however, where we sampled at 24 h intervals, it is unknown what happened in-between the first 24 h interval or any of the other subsequent intervals. A shorter time interval could, for instance, show an initial increase in LA-MRSA prevalence before a decrease occurs. Other bacteria, such as ingested *Aeromonas caviae*, initially increase in numbers the first two days after being fed to house flies, followed by a sharp decrease^42^. Ingested *Bacillus anthracis* shows a similar trend, although within a shorter time span, with *B. anthracis* initially increasing up to 10 h post-ingestion, followed by a sharp decline with the remaining bacteria lingering up to 24 h post-ingestion^43^.

According to the LA-MRSA proportion- and time-to-event analysis data from our experiment, on average, 7% of stable flies and 26.5% of house flies leaving the LA-MRSA contaminated pig farm tested positive for LA-MRSA. For the stable flies, the percentage of flies with live LA-MRSA bacteria on average was 5.4% after 24 h, 3.5% after 48 h, 3.1% after 72 h and 0.4% after 96 h. For the house flies, the percentage of flies with live LA-MRSA bacteria on average was 7.8% after 24 h, 4.3% after 48 h, 2.2% after 72 h and 0% after 96 h.

The distance estimations derived from the Quarterman et al.^38^, Schoof et al.^39^ and Chakrabarti et al.^40^ studies indicate that almost all pig farms or residential addresses in Denmark can be reached by stable- and house flies within a 24 h timeframe where, according to our time-to-event analysis, still a substantial proportion of flies would carry live LA-MRSA bacteria. Although the distance estimates derived from the Taylor et al.^37^ study are shorter, still a substantial proportion of Danish pig farms and residential addresses would lie within reach of a stable- or house fly which could potentially still be carrying live LA-MRSA bacteria. It is important to note that a fly leaving a farm has a myriad of directions it could fly towards and is not bound to fly to its nearest neighbouring pig farm or a residential address. However, given the hematophagous and saprophytic foraging behaviours of stable- and house flies, respectively, as well as the selective flight behaviour resulting from olfactory-^44^ and visual cues^45^, pig farms, humans and residential addresses^21^ would be attractive target locations for foraging or oviposition. We would like to stress that our dispersal distance estimates simplify a real world scenario and should therefore not be used in simulation spread models for LA-MRSA or models that assess the zoonotic risk of fly-borne LA-MRSA transmission for people living near pig farms. The dispersal distance estimates we derived from the literature, combined with our own LA-MRSA survival measurements do however indicate that stable- and houseflies as a potential transmission route could help explain the observed rapid spread of LA-MRSA amongst Danish pig farms and its associated zoonotic spread. A future study showing precise measurements of stable- and house fly dispersal is needed before our LA-MRSA contamination and survival rates can be used in such models.

A study by Rosen et al.^46^ showed that exposure to aerosol MRSA particles is a viable transmission route for piglets, whereas Angen et al.^47^ found that increased carriage of LA-MRSA amongst volunteers on a pig farm was dependent on the airborne concentration of MRSA, rather than direct contact with the pigs. LA-MRSA from dust samples collected inside pig farms was found to have a half-life of 5 days, with a 99% die-off rate of 66 days^48^. This can pose a risk to humans, not just inside the farm, but also in the surrounding environment, as antibiotic-resistant bacteria can spread downwind of a pig farm facility^9^ in levels that could potentially pose a risk to human health^10^. However, unlike airborne LA-MRSA dust, flying insect-borne transmission would be much less dependent on wind conditions and more directed due to the foraging- and flight behaviour of both stable- and house flies. With their saprophytic foraging behaviour, house flies could pose a potential health risk to humans, especially those with skin lesions or lacerations, if that fly is carrying LA-MRSA bacteria.

The environmental contamination experiment showed that no direct contact with a pig or the farm’s interior surfaces was required for a fly to collect LA-MRSA bacteria. We found only small differences in LA-MRSA prevalence between the cultivated caged house flies exposed to the air in the stables for 48 h and the free-roaming wild flies in each room. This observation supports previous studies which found that both piglets^46^ and pig farm volunteers^47^ can become carriers of LA-MRSA through environmental exposure to airborne LA-MRSA dust particles inside the farm. It is possible that the experimental setup, where we used a 1mm^2^ plastic mesh insect net to construct the cages, had an effect on the observed difference between the two groups for each room. It is also possible that LA-MRSA dust^49^ accumulated on the cages or that caged cultivated house flies were able to touch LA-MRSA positive wild free-roaming flies through the mesh, increasing the LA-MRSA prevalence amongst the caged cultivated house flies. It is however unlikely that a significant proportion of the observed LA-MRSA prevalence amongst the caged cultivated flies can be attributed to direct contact with wild free-roaming flies. The similar observed proportions of LA-MRSA positive flies between the free-roaming wild flies and caged cultivated house flies support this. If none of the caged cultivated house flies became LA-MRSA positive due to environmental exposure to airborne LA-MRSA, the required level of contamination needed from direct contact with free-roaming wild flies to achieve this would likely have resulted in a much higher prevalence of LA-MRSA amongst wild free-roaming flies than we observed.

One significant limitation of this present study is that flies were only categorized as testing positive or negative for LA-MRSA. By not quantifying the bacteria present on the flies that tested positive, it is not possible in this experimental setup to infer whether the quantity of live LA-MRSA bacteria present on a fly correlates with the observed survival time. More importantly, we did not investigate if a fly is actually capable of transmitting LA-MRSA to a pig, pig farm or human, and if so, in what quantity live LA-MRSA bacteria need to be present for this transmission to be possible. As a result of this, we are not able to conclude that stable- and house flies are mechanical vectors. However, we do consider it a valid assumption that mechanical transmission of LA-MRSA by both stable- and house flies is possible, considering the previous findings on the vector potential of flies with other pathogens and our own findings. This assumption is further strengthened by the simulation models on the known pathways in the spread of LA-MRSA which are not able to explain the entire observed spread in Denmark^5^. Furthermore, it is important to note that we consider stable- and house flies to be potential mechanical vectors and not biological vectors, which is supported by our Kaplan–Meier time-to-event curves (Fig. 2) as these suggest that the LA-MRSA bacteria do not replicate on the flies.

Due to the two species of flies not being collected or housed separately, there is the possibility that one species cross-contaminated the other either in the butterfly nets or cylindrical containers, creating a confounding effect on LA-MRSA prevalence of the two species. Our binary logistic regression model does however indicate a significantly higher likelihood of a house fly testing positive to LA-MRSA compared to a stable fly. In addition, the alternative parametric survival analysis that we eventually discarded due to the data fitting the Weibull model poorly, also found a strong effect for fly species. We believe that if this cross-contamination had a strong confounding effect, these models would not have found any significant differences between the two species. We do however find it important to mention this potential form of bias. It is also a possibility that one species could have shed the LA-MRSA significantly faster than the other, but kept being re-contaminated with LA-MRSA bacteria by the other species. This form of bias cannot be excluded as we did not separate the two species or incorporated the number of LA-MRSA bacteria per fly in this study.

Another limitation of the study is that not much is known about the dispersal rates and migration numbers of stable- and house flies from a pig farm, which in turn would likely have a significant influence on their transmission potential of LA-MRSA. A few studies confirm that stable flies and house flies migrate between farms, but specific estimates of how frequent migration between farms takes place and in what quantities is currently lacking. A study by Beresford & Sutcliffe^22^ found that stable flies in Ontario, Canada, recolonize local farms in late spring and early summer following dispersal from their natal source refuge farms when the farm’s carrying capacity is exceeded. Genetic analysis of house fly populations in rural livestock farms and urban locations in Kansas, USA, indicated that house flies frequently migrate between these areas in considerable quantities^40^. During our study, we briefly attempted to catch stable- and house flies outside the farm and the surrounding area. Even though we were only successful within a 20 m radius of the pig farm, some of these flies did test positive for LA-MRSA, indicating that LA-MRSA contaminated fly populations are not confined to the farm’s interior.

Our results suggest that it is potentially temporally possible for stable- and house flies to transport live LA-MRSA bacteria from LA-MRSA positive pig farms to neighbouring pig farms or residential addresses in Denmark, and that stable- and house flies thus should be considered as potential mechanical vectors of LA-MRSA. However, before stable- and house flies can be considered as viable mechanical vectors, further studies are needed to measure the quantities of LA-MRSA bacteria being transported and whether this is a viable source of infection for humans or uncontaminated pig farms. Hald et al.^50^ showed that the application of fly screens on the windows of broiler farms significantly reduced the number of *Campylobacter* spp.-positive broiler flocks in these farms, indicating that flies can act as vectors. Should stable- and house flies be capable of transmitting LA-MRSA through direct contact with pigs or the farm’s interior, impeding flies from exiting or entering a pig farm might prove effective at reducing this potential transmission pathway. If stable- and house flies are found capable of being mechanical vectors, it will also be necessary to quantify migration numbers between farms and from farms to humans in order to assess the associated risk of this transmission pathway and the compare this risk with other known transmission pathways.
